# Impact of Smoking-Related Chronic Obstruction Pulmonary Disease on Mortality of Invasive Ductal Carcinoma Patients Receiving Standard Treatments: Propensity Score-Matched, Nationwide, Population-Based Cohort Study

**DOI:** 10.3390/cancers13153654

**Published:** 2021-07-21

**Authors:** Jia-Qiang Zhang, Tsai-Mu Cheng, Wei-Chun Lin, Kuo-Chin Chiu, Szu-Yuan Wu

**Affiliations:** 1 Department of Anesthesiology and Perioperative Medicine, Henan Provincial People’s Hospital, People’s Hospital of Zhengzhou University, Zhengzhou 450052, China; jqzhang@henu.edu.cn; 2 Centers for Regional Anesthesia and Pain Medicine, Wan Fang Hospital, Taipei Medical University, Taipei 110, Taiwan; 3 The Ph.D. Program for Translational Medicine, College of Medical Science and Technology, Taipei Medical University, Taipei 110, Taiwan; tmcheng@tmu.edu.tw; 4 Division of Chest Medicine, Department of Internal Medicine, Lo-Hsu Medical Foundation, Lotung Poh-Ai Hospital, Yilan 256, Taiwan; 973002@mail.pohai.org.tw; 5 Department of Food Nutrition and Health Biotechnology, College of Medical and Health Science, Asia University, Taichung 413, Taiwan; 6 Big Data Center, Lo-Hsu Medical Foundation, Lotung Poh-Ai Hospital, Yilan 256, Taiwan; 7 Division of Radiation Oncology, Lo-Hsu Medical Foundation, Lotung Poh-Ai Hospital, Yilan 256, Taiwan; 8 Department of Healthcare Administration, College of Medical and Health Science, Asia University, Taichung 413, Taiwan; 9 Cancer Center, Lo-Hsu Medical Foundation, Lotung Poh-Ai Hospital, Yilan 256, Taiwan; 10 Graduate Institute of Business Administration, Fu Jen Catholic University, Taipei 242062, Taiwan

**Keywords:** breast intraductal carcinoma, COPD, COPDAE, cigarette smoking, survival

## Abstract

**Simple Summary:**

This study is the first to estimate the impact of smoking-related chronic obstructive pulmonary disease (COPD) on invasive ductal carcinoma (IDC) patients receiving standard treatments. Smoking-related COPD was not a significant independent risk factor for all-cause mortality in women with stage I–III IDC receiving standard treatments. The frequency of hospitalization for COPD with at least one acute exacerbation within one year before breast surgery was highly associated with high mortality for women with IDC receiving standard treatments.

**Abstract:**

Purpose: the survival effect of smoking-related chronic obstructive pulmonary disease (COPD) and COPD with acute exacerbation (COPDAE) is unclear for patients with invasive ductal carcinoma (IDC) receiving standard treatments. Methods: we recruited women with clinical stage I–III IDC from the Taiwan Cancer Registry Database who had received standard treatments between 1 January 2009 and 31 December 2018. The time-dependent Cox proportional hazards model was used to analyze all-cause mortality. To reduce the effects of potential confounders when all-cause mortality between Groups 1 and 2 were compared, 1:2 propensity score matching (PSM) was performed. We categorized the patients into two groups based on COPD status to compare overall survival outcomes: Group 1 (current smokers with COPD) and Group 2 (nonsmokers without COPD group). Results: PSM yielded 2319 patients with stage I–III IDC (773 and 1546 in Groups 1 and 2, respectively) eligible for further analysis. In the multivariate time-dependent Cox regression analyses, the adjusted hazard ratio (aHR; 95% confidence interval (CI)) of all-cause mortality for Group 1 compared with Group 2 was 1.04 (0.83–1.22). The aHRs (95% CIs) of all-cause mortality for ≥1 hospitalization for COPDAE within one year before breast surgery was 1.51 (1.18–2.36) compared with no COPDAE. Conclusion: smoking-related COPD was not a significant independent risk factor for all-cause mortality in women with stage I–III IDC receiving standard treatments. Being hospitalized at least once for COPDAE within one year before breast surgery is highly associated with high mortality for women with IDC receiving standard treatments. The severity of smoking-related COPD before treatments for breast cancer might be an important prognostic factor of survival. Thus, the information of the severity of COPD before treatment for breast cancer might be valuable for increasing the survival rate in treatment of breast cancer, especially in the prevention of progress from COPD to COPDAE.

## 1. Introduction

Smoking-related chronic obstructive pulmonary disease (COPD) and COPD with acute exacerbation (COPDAE) may indicate severe lung inflammation or poor heart function, and may be a surrogate marker of pulmonary or cardiac function [1,2,3,4]. COPD or COPDAE may trigger major adverse cardiac events (MACE) [5]. The risk of MACE increases substantially following COPDAE [5]. Prevention of such MACE is a critical goal in COPD management to avoid COPDAE [5]. Many studies have indicated an increased risk of breast cancer in smokers [6,7,8,9,10]. The relationship between cigarette smoking and breast cancer is complicated.

No study has analyzed the severity of COPD and survival outcomes for women with invasive ductal carcinoma (IDC) receiving curative standard treatments (breast surgery followed by adjuvant chemotherapy, anti-human epidermal growth factor receptor 2 [HER2] tyrosine kinase inhibitors, hormone therapy, or adjuvant irradiation according to the National Comprehensive Cancer Network [NCCN] guidelines [11]), although many studies have concluded that having previously experienced MACE causes poor survival in women with breast cancer undergoing breast surgery followed by systemic chemotherapy or adjuvant radiotherapy [12,13,14,15,16,17,18]. The cardiotoxicity or lung injury of chemotherapy and radiotherapy can be more severe in women who have experienced MACE or have underlying comorbidities such as COPD [12,13,14,15,16,17,18,19,20]; having had MACE or having COPD or COPDAE may be risk factors for all-cause mortality for women with breast cancer receiving standard treatments.

Therefore, we assessed whether the severity of smoking-related COPD (COPD, or hospitalization for COPDAE before standard treatments for patients with breast cancer) is an independent prognostic factor of overall survival (OS) in patients with IDC undergoing breast surgery followed by adjuvant treatments based on NCCN guidelines [11]. The severity of COPD before breast cancer treatment may be an important prognostic factor for survival. Therefore, understanding the severity of COPD before breast cancer treatment may be of great significance to improve the survival rate of breast cancer treatment, especially to prevent COPD from progressing to COPDAE.

## 2. Patients and Methods

### 2.1. Study Population

We enrolled patients from the Taiwan Cancer Registry Database (TCRD) with a diagnosis of American Joint Committee on Cancer (AJCC) clinical stage I–III breast IDC between 1 January 2009 and 31 December 2018. The index date was the date of breast surgery, and the follow-up duration was from the index date to 31 December 2019. The TCRD contains detailed cancer-related information of patients, including the stage, cigarette smoking habit, treatment modalities, pathologic data, irradiation doses, hormone receptor (HR) status, HER2 status, radiotherapy, and chemotherapy regimens used [21,22,23,24,25,26]. The study protocols were reviewed and approved by the Institutional Review Board of the Tzu-Chi Medical Foundation (IRB109-015-B).

### 2.2. Inclusion and Exclusion Criteria

The diagnoses of the enrolled patients were confirmed after reviewing their pathological data, and the women with newly diagnosed IDC were confirmed to have no other cancers or distant metastases. The women were included if they had received an IDC diagnosis, were 20 years old or older, and had clinical stage I–III (AJCC, 8th edition) without metastasis. Patients were excluded if they had a history of cancer before the IDC diagnosis date, unknown pathologic types, missing sex data, unclear staging, and non-IDC histology. In addition, patients with nonstandard adjuvant breast radiotherapy (in contrast with standard adjuvant radiotherapy, consisting of irradiation to both the chest wall/whole breast and regional nodes with a minimum of 50 Gy), neoadjuvant chemotherapy, unclear differentiation of tumor grade, missing HR status, missing HER2 status, or unclear staging were excluded. Adjuvant treatments such as adjuvant radiotherapy, adjuvant chemotherapy, hormone therapy, or target therapy were allowed based on NCCN guidelines in Taiwan [11]. We also excluded patients with unclear surgical procedures, ill-defined nodal surgery, unclear Charlson comorbidity index (CCI), or unclear differentiation from our cohort. HR positivity was defined as ≥1% of tumor cells demonstrating positive nuclear staining through immunohistochemistry [27], and HER2 positivity was defined as an immunohistochemistry score of 3+ or a fluorescence in situ hybridization ratio of ≥2 [28,29].

After applying the inclusion and exclusion criteria, we enrolled 2319 women with AJCC clinical stage I–III IDC who had received breast surgery and a sentinel lymph node biopsy (SLNB) or axillary lymph node dissection (ALND), and divided them into two groups based on their smoking-related COPD status to compare all-cause mortality: Group 1 (current smokers with smoking-related COPD before breast surgery) and Group 2 (nonsmokers without COPD before breast surgery). We also estimated the survival outcome of the severity of smoking-related COPD (frequency of hospitalization for COPDAE with 0 or ≥1 hospitalizations within one year before the index date) and patients with stage I–III IDC undergoing breast surgery. Breast surgery including partial (breast-conserving surgery) and total mastectomy were included in our study. Breast-conserving therapy refers to breast-conserving surgery (BCS; i.e., lumpectomy) typically followed by moderate-dose radiation therapy (RT) to eradicate any microscopic residual disease. The incidence of comorbidities was scored using the CCI [30,31]. MACE refer to a set of comorbidities frequently used in cardiovascular research [32,33] and, herein, consist of a composite of nonfatal stroke, nonfatal myocardial infarction, cardiovascular event, and admission for heart failure [34,35,36]. MACE, hypertension, diabetes, COPD, hyperlipidemia, and chronic kidney disease (CKD) were excluded from the CCI scores to prevent repetitive adjustment in multivariate analysis. Only comorbidities observed within 12 months before the index date were included; they were coded and classified according to the International Classification of Diseases, 10th Revision, Clinical Modification (ICD-10-CM) codes at the first admission, or after more than two repetitions of a code were issued at outpatient department visits.

Current smokers were recorded by the national professional cancer registrar in the TCRD, which means an adult who has smoked 100 cigarettes in his or her lifetime and who currently smokes cigarettes. The number of current smokers were recorded by these national professional cancer registrars, certified by the Taiwan Cancer Registry. There is no record for abstinence, because current smokers were defined as a patient with breast IDC who currently smokes cigarettes at the index date. Non-smokers were recorded by these national professional cancer registrars in the TCRD, which means an adult who has never smoked cigarettes in his or her lifetime. The non-smokers were recorded by these national professional cancer registrars, certified by the Taiwan Cancer Registry. COPD group were identified as observed within 12 months before the index date; they were used as the main diagnosis code according to ICD-10-CM codes for the first admission, or the main diagnosis code for the two outpatient visits would be classified in the COPD group. Hospitalization of COPDAE defined within 12 months before the index date were included; they were coded and classified according to the ICD-10-CM codes at the first admission.

### 2.3. Propensity Score Matching and Covariates

To reduce the effects of potential confounders when all-cause mortality between Groups 1 and 2 were compared, 1:2 propensity score matching (PSM) was performed with a caliper of 0.2 for the following variables: age, menopausal status, CCI score, differentiation, AJCC clinical stage, adjuvant chemotherapy, adjuvant radiotherapy, HR status, Her-2 status, nodal surgery, types of breast surgery, history of MACE, hypertension, diabetes, hyperlipidemia, alcohol use, drug abuse, and CKD [37]. There were no eligible patients that could not be matched in our study. A time-dependent Cox regression model was only utilized for HRs related to time-dependent variables, namely treatments (chemotherapy and radiotherapy). A Cox regression model was used to regress all-cause mortality on different COPD statuses, with a robust sandwich estimator used to account for clustering within matched sets [38]. Multivariate time-dependent Cox regression analyses were performed to calculate hazard ratios to determine whether the factors of COPD status, frequency of hospitalization for COPDAE within one year before the index date, age, menopausal status, CCI score, differentiation, AJCC clinical stage, adjuvant chemotherapy, adjuvant radiotherapy, HR status, Her-2 status, nodal surgery, types of breast surgery, MACE, hypertension, diabetes, COPD, hyperlipidemia, alcohol use, drug abuse, and CKD were potential independent predictors of all-cause mortality. Potential predictors were controlled for in the analysis (Table 1), and all-cause mortality was the primary endpoint in both groups.

### 2.4. Statistics

Continuous variables are expressed as mean ± SD. Comparisons among the two groups were conducted using independent *t*-tests for continuous variables and a Chi-square test for categorical variables. The Mann–Whitney U test is used to compare differences of follow-up time between the two groups. We have used a Gray’s test to produce the two *p* values for adjuvant RT and adjuvant chemotherapy in Table 1. After adjustment for confounders, all analyses were performed using SAS version 9.3 (SAS Institute, Cary, NC, USA). In a two-tailed Wald test, *p* < 0.05 was considered significant. OS was estimated using the Kaplan–Meier method, and differences among non-COPD, COPD, and hospitalization for COPDAE were determined using the stratified log-rank test to compare survival curves (stratified according to matched sets) [39].

## 3. Results

### 3.1. Propensity Score Matching and Study Cohort

PSM yielded 2319 patients with stage I–III IDC (773 and 1546 in Groups 1 and 2, respectively) eligible for further analysis. Table 1 summarizes their clinicodemographic characteristics. Age, menopausal status, CCI score, differentiation, AJCC clinical stage, adjuvant chemotherapy, adjuvant radiotherapy, HR status, Her-2 status, nodal surgery, types of breast surgery, MACE, hypertension, diabetes, hyperlipidemia, alcohol use, drug abuse, and CKD were similar between the two groups due to PSM. Follow-up duration and hospitalization for COPDAE within one year before breast surgery was inconsistent between the two groups (Table 1).

### 3.2. Prognostic Factors of All-Cause Mortality after Multivariate Cox Regression Analyses

Multivariate Cox regression analysis indicated that hospitalization for COPDAE within one year before the index date, old age, high CCI, advanced AJCC clinical stage, high grade of differentiation, and history of MACE were associated with poor OS (Table 2). No significant differences were observed in menopausal status, adjuvant chemotherapy, adjuvant radiotherapy, HR status, Her-2 status, nodal surgery, types of breast surgery, hypertension, diabetes, COPD, hyperlipidemia, alcohol use, drug abuse, or CKD (Table 2). The adjusted hazard ratio (aHR; 95% CI) of all-cause mortality for Group 1 compared with Group 2 was 1.04 (0.83–1.22; *p* = 0.782). The aHRs (95% CIs) of all-cause mortality for ≥1 hospitalization for COPDAE within one year before breast surgery was 1.51 (1.18–2.36; *p* = 0.002) compared with no COPDAE in patients with stage I–III IDC undergoing breast surgery. Moreover, aHRs (95% CIs) of all-cause mortality for the age groups of 51–60 years, 61–70 years, and >70 years; CCI 1 and ≥2; AJCC clinical stage II and III; differentiation grade II and III; and history of MACE were 1.54 (1.12–2.13), 2.32 (1.67–3.21), 4.92 (3.50–6.90); 1.52 (1.24–2.12), 1.85 (1.26–2.70); 1.22 (1.06–1.93) and1.47 (1.13–1.85); 1.03 (1.01–1.47) and 1.08 (1.07–1.35); and 1.31(1.14–2.25) respectively, compared with age ≤ 50 years; CCI = 0; AJCC clinical stage I; differentiation grade I; and no history of MACE, respectively.

### 3.3. Kaplan–Meier OS among Non-COPD, COPD, and Hospitalization for COPDAE

**Figure 1:**
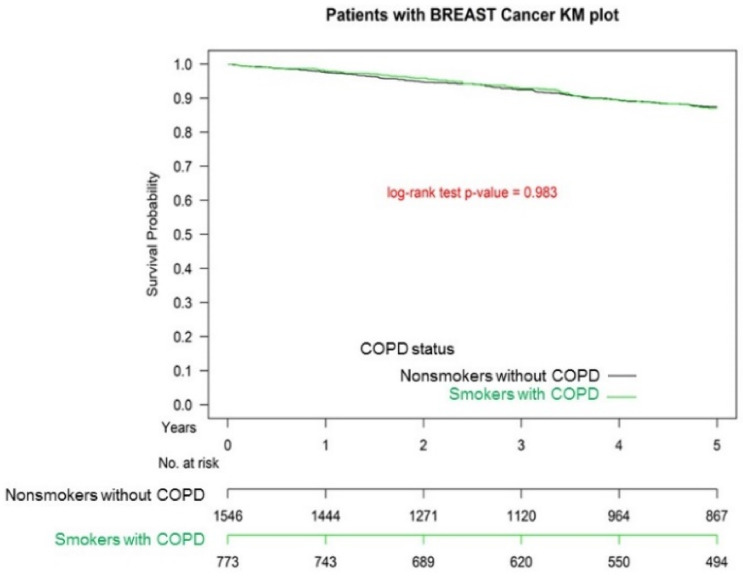
Kaplan–Meier survival curves of patients with invasive ductal carcinoma with or without smoking-related chronic obstructive pulmonary disease (COPD) before breast surgery.

**Figure 2:**
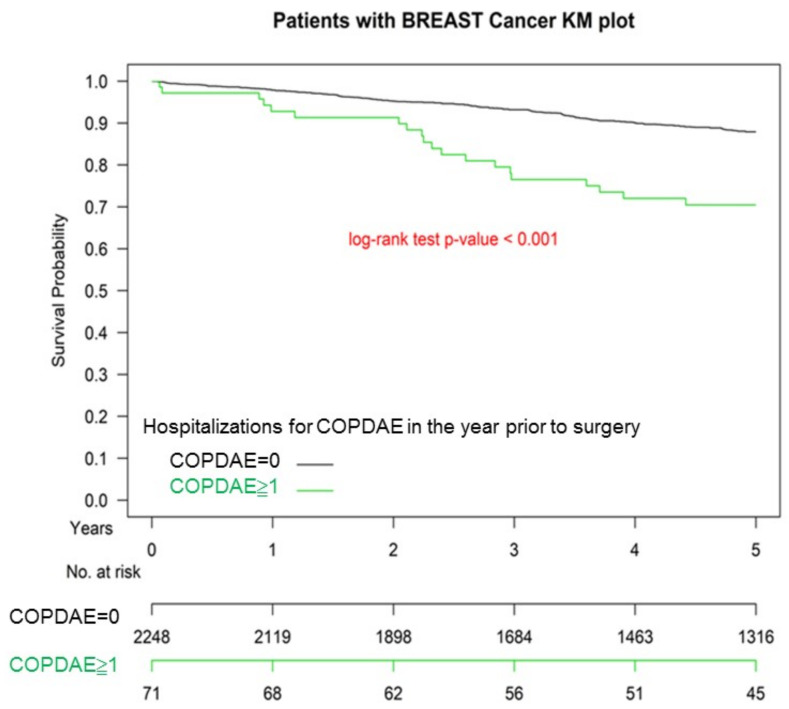
Kaplan–Meier survival curves of patients with invasive ductal carcinoma with frequency of hospitalization for COPDAE within 1 year before breast surgery. COPDAE, chronic obstruction pulmonary disease with acute exacerbation.

Figure 1 presents the Kaplan–Meier survival curves for the two groups. The OS was not significantly different between the two groups (*p* = 0.983). The OS of patients with IDC with ≥1 hospitalization for COPDAE within one year before breast surgery was poorer than that for those with 0 hospitalizations for COPDAE (*p* < 0.001) (Figure 2).

## 4. Discussion

The mechanism is largely unclear by which COPD increases cancer risk [40]. Smoking is a common shared risk factor for COPD and solid organ cancers (including breast cancer) [40]. However, even after adjusting for smoking, a significant relationship between COPD and cancer was observed [40]. Among patients with breast cancer, comorbidities in general and specifically cardiovascular diseases, COPD, diabetes, and venous thromboembolism negatively affect OS [41]. Thus, smoking related COPDAE might contribute to poor OS in patients with breast cancer receiving breast surgery, although no study has analyzed this. The severity of cigarette smoking-related COPD might be proportional to the severity of poor pulmonary function [1,2,3,4] or poor cardiac function [5] attributable to the higher mortality due to the progression of toxicity by treatments of IDC. Ours is the first study to evaluate whether the severity of smoking-related COPD is a significant prognostic factor of OS in the patients with IDC receiving standard treatments.

Because of PSM, all potential covariates associated with the OS of breast cancer patients receiving treatments were well-matched between the two groups. Our study is the first head-to-head PSM study to estimate the severity of current smoking-related COPD or COPDAE for patients with IDC undergoing breast surgery and standard adjuvant treatments based on NCCN guidelines [11].

Our data indicated no significant association of cigarette smoking-related COPD and OS for women with IDC receiving standard treatments. No study has analyzed smoking-related COPD as a risk factor for all-cause mortality in patients with breast cancer receiving treatments, even though cigarette smoking is significantly associated with a poor prognosis in women diagnosed with breast cancer [42]. Our study is the first study to show that current smoker-related COPD was not a significant prognostic factor for women with IDC receiving standard treatments. We found that hospitalization for COPDAE within one year before breast treatment was an independent prognostic factor of OS. The severity of smoking-related COPD, evident in occurrences such as hospitalization for COPDAE (the same as the Global Initiative for Chronic Obstructive Lung Disease [GOLD] Classification 3–4) [43], was a significant independent prognostic factor of mortality for women with IDC receiving standard treatments. This may be because severe COPDAE with poor pulmonary or cardiac function worsened the OS in patients with IDC receiving standard treatments, probably due to radiation-induced intolerable lung injury (RILI) or treatment-induced cardiotoxicity [12,13,14,15,16,17,18,19,20].

Preexisting COPD and female sex are associated with an increased risk of radiation pneumonitis in patients with breast cancer undergoing radiotherapy [19,20]. In addition, not only COPD-related RILI but also patient-related factors may increase the risk of radiation-induced cardiotoxicity, including coronary heart disease, one of the MACE [12,13]. Preexisting cardiovascular disease (one of the MACE) may increase the radiation induced cardiac toxicity (RICT) [12,13]. In addition, cancer patients receiving chemotherapy have an increased risk of cardiovascular complications, and the risk is even greater with a history of heart disease [44,45]. Anthracycline and anthracycline-like agents [14,15,16,17,18,46] and HER2-targeting agents, such as trastuzumab [47,48] and fluoropyrimidines [44], are anticancer agents that are well known to be associated with cardiac toxicity. Concomitant chronic cardiac disorders such as MACE are frequent in patients with COPD [49]. Risk factors for anthracycline cardiac toxicity include female sex, COPD, and MACE [14,15,16,17,18,46,49]. Therefore, COPD or MACE may lead to more severe cardiotoxicity after systemic therapy in patients with breast cancer [14,15,16,17,18,44,46,47,48,49]. In our study, a history of MACE associated with higher risk of all-cause mortality after multivariate analysis echoes the above findings (Table 2) [14,15,16,17,18,46]. Thus, hospitalization for COPDAE within one year before standard treatments for IDC might indicate that poorer pulmonary (RILI) or poor cardiac function (RICT or chemotherapy induced cardiac toxicity) [1,2,3,4,5] contributed to worse survival compared with those without COPDAE (Table 2).

In our study, MACE and preexisting COPDAE, but not hypertension, diabetes, hyperlipidemia, COPD, alcohol use, drug abuse, or CKD, were significant prognostic factors of all-cause mortality in patients with IDC receiving standard treatments (Table 2). In a previous study, preoperative MACE (adjusted odds ratio, 1.21; 95% CI, 1.14–1.29) were found to be a prognostic marker for perioperative 30-day morbidity and mortality for cancer patients [50]. In our study, MACE were an independent risk factor for all-cause mortality for patients with IDC receiving standard treatments, in accord with other studies [12,13,14,15,16,17,18]. MACE seem to be more predictive for OS of patients with IDC receiving standard treatments than hypertension, diabetes, COPD, hyperlipidemia, alcohol use, drug abuse, or CKD (Table 2). The other poor prognostic factors of all-cause mortality for patients with IDC undergoing breast surgery and adjuvant treatments according to NCCN guidelines were old age, high CCI, high grade differentiation, and advanced clinical stages (Table 2), in accordance with previous studies [51,52,53,54,55].

Neoadjuvant chemotherapy is associated with high rates of clinical response and a greater likelihood of facilitating cosmetically acceptable surgery [22,24,56]. For example, patients who were not candidates for breast conservation may become eligible after neoadjuvant chemotherapy [22,24,56]. Most patients with early stage (AJCC stage I–II) breast cancer receiving breast surgery would not need neoadjuvant chemotherapy in our study, compatible with other studies [22,24,56]. Thus, fewer patients received neoadjuvant chemotherapy in our study, because there were more than 75% stage I–II early stage breast cancers in the current study (Table 1). Additionally, various regimens and different courses of neoadjuvant chemotherapy would cause too many covariates in our analysis. Moreover, the response rates (complete response, partial response, stationary disease, and progression of disease) of neoadjuvant chemotherapy are strongly associated with survival for women with breast cancer receiving neoadjuvant chemotherapy [23,25]. Therefore, if we consider including neoadjuvant chemotherapy for fewer patients with early breast IDC, we need to consider additional covariates including response rate (complete response, partial response, stationary disease, and progression of disease), regimens of chemotherapy, and courses (four, six, or eight courses) of neoadjuvant chemotherapy in our study [22,23,24,25,56]. However, too many covariates in a multivariable model may cause the problem of overfitting [57], especially in a small sample size for neoadjuvant chemotherapy, as in our study. In addition, a regression model containing too many variables would result in overspecified bias [58].

The strength of our study was that it was the first and largest cohort study to estimate the survival outcomes of current smoking-related COPD compared with nonsmokers without COPD among patients with IDC undergoing breast surgery and adjuvant treatments based on NCCN guidelines [11]. PSM led to comparable covariates between groups, and no selection bias was noted (Table 1). No prior study has estimated the impact of COPD and hospitalization for COPDAE in breast cancer patients receiving standard treatments, and all of the prognostic factors were evaluated. In our study, the poor prognostic factors of OS in these patients with breast cancer were similar, such as CCI ≥ 1, moderate to poor differentiation, advanced clinical stages II–III, and old age (Table 2), and were in accord with previous studies [51,52,53,54,55]. Until now, there has been no evidence for proving the risk of all-cause death for COPDAE before treatment of breast cancer, and resulting worse survival. This is the first study to demonstrate with real world data that COPD was not associated with overall survival; 1+ hospitalizations for COPDAE in the year prior to surgery was associated with an increased risk of death. Because 1+ hospitalizations for COPDAE in the year prior to surgery was associated with an increased risk of death, well-controlled COPD disease prevention from COPDAE is valuable for breast cancer survival in future clinical practice. In addition, 1+ hospitalizations for COPDAE in the year prior to surgery was associated with an increased risk of death, and should be considered in prospective clinical trials for breast cancer research.

There are some limitations in our study. First, all IDC patients are from Asian populations; therefore, our results should be carefully extrapolated to non-Asian populations. However, there is no evidence that there is a difference in the oncology results of IDC patients receiving standard treatment between Asian and non-Asian populations. Second, the diagnosis of all comorbidities is based on the ICD-10-CM code. The Taiwan Cancer Registry Administration randomly reviewed medical records and interviewed patients to verify the accuracy of the diagnosis. If improper behavior or discrepancies are found, hospitals with abnormal charges or practices will be audited and severely punished. However, in order to obtain critical information about population specificity and disease occurrence, large-scale randomized trials must be conducted to compare carefully selected patients receiving appropriate treatment. Finally, the TCRD does not contain information on socioeconomic status, body mass index, or eating habits, all of which may be risk factors for death in IDC patients. However, given the magnitude and statistical significance of the effects observed in this study, these limitations are unlikely to affect the conclusions.

## 5. Conclusions

No association of survival outcomes was observed in women with IDC undergoing breast surgery who had current smoking-related COPD or who were nonsmokers but had COPD. Hospitalization for COPDAE within one year before breast surgery was found to be an independent risk factor for OS for women with IDC receiving standard treatments.

## Figures and Tables

**Table 1:** Characteristics of patients with invasive ductal carcinoma with or without smoking-related COPD before breast surgery after propensity score matching.

Variables	Nonsmokers without COPD		Smokers with COPD		*p*-Value
	*N* = 1546		*N* = 773		
		*N*, %		*N*, %	
Age (mean ± SD)	(58.37 ± 12.59)		(58.83 ± 12.37)		0.404
Age (years)					0.467
≤50	396	25.61%	209	27.04%	
51–60	444	28.72%	212	27.43%	
61–70	414	26.78%	191	24.71%	
>70	292	18.89%	161	20.83%	
CCI score					0.310
0	1417	91.66%	694	89.78%	
1	30	1.94%	20	2.59%	
≥2	99	6.40%	59	7.63%	
CCI score (mean ± SD)	(0.16 ± 0.59)		(0.21 ± 0.70)		0.108
Menopausal status					0.320
Postmenopausal	996	64.42%	462	59.77%	
Premenopausal	550	35.58%	311	40.23%	
Her2 status					0.422
Negative	1259	81.44%	618	79.95%	
Positive	287	18.56%	155	20.05%	
Nodal surgery					0.891
SLNB	1082	69.99%	543	70.25%	
ALND	464	30.01%	230	29.75%	
AJCC clinical stage					0.782
I	801	51.81%	408	52.78%	
II	376	24.32%	193	24.97%	
III	369	23.87%	172	22.25%	
Hormone receptor					0.792
Negative	345	22.32%	177	22.90%	
Positive	1201	77.68%	596	77.10%	
Breast surgery					0.726
Total mastectomy	228	14.75%	119	15.39%	
Breast-conserving surgery	1318	85.25%	654	84.61%	
Differentiation					0.692
I	228	14.75%	119	15.39%	
II	731	47.28%	351	45.41%	
III	587	37.97%	303	39.20%	
Adjuvant chemotherapy					0.177
No	756	48.90%	403	52.13%	
Yes	790	51.10%	370	47.87%	
Adjuvant radiotherapy					0.812
No	228	14.75%	119	15.39%	
Yes	1318	85.25%	654	84.61%	
MACE history					0.322
No	1114	72.06%	541	69.99%	
Yes	432	27.94%	232	30.01%	
Hyperlipidemia					0.566
No	1138	78.16%	589	76.20%	
Yes	318	21.84%	184	23.80%	
Hypertension					0.664
No	965	66.28%	502	64.94%	
Yes	491	33.72%	271	35.06%	
Diabetes					0.645
No	1164	79.95%	610	78.91%	
Yes	292	20.05%	163	21.09%	
Chronic kidney disease					1.000
No	1441	98.97%	765	98.97%	
Yes	15	1.03%	8	1.03%	
Alcohol use					0.492
No	1268	82.02%	618	79.94%	
Yes	278	17.98%	155	20.06%	
Drug abuse					0.284
No	1500	97.02%	743	96.12%	
Yes	46	2.98%	30	3.88%	
Frequency of hospitalization for COPDAE within 1 year before breast surgery					<0.001
0	1546	100.00%	702	90.82%	
1	0	0.00%	39	5.05%	
≥2	0	0.00%	32	4.14%	
Follow-up (All-cause mortality) Years, Median (IQR, Q1–Q3)	7.21 (3.53–12.06)		5.79 (2.59–9.81)		<0.001
Follow-up (Did not die) Years, Median (IQR, Q1–Q3)	5.41 (3.49–11.93)		5.15 (2.56–9.71)		0.788

IQR, interquartile range; SD, standard deviation; AJCC, American Joint Committee on Cancer; CCI, Charlson comorbidity index; ALND, axillary lymph node dissection; COPD, chronic obstructive pulmonary disease; COPDAE, COPD with acute exacerbation; MACE, major adverse cardiovascular events.

**Table 2:** Cox proportional hazards analysis of all-cause mortality for patients with invasive ductal carcinoma with or without smoking-related COPD before breast surgery.

Variables	Crude HR (95% CI)		Adjusted HR * (95% CI)		*p*-Value
COPD status (ref: non-COPD)					
COPD	1.07	(0.88–1.31)	1.04	(0.83–1.22)	0.782
Frequency of hospitalization for COPDAE within 1 year before breast surgery (ref: 0)					
≥1	2.86	(1.78–3.54)	1.51	(1.18–2.36)	0.002
Age (years, ref: ≤50)					
51–60	1.71	(1.23–2.31)	1.54	(1.12–2.13)	0.004
61–70	2.51	(1.85–3.39)	2.32	(1.67–3.21)	<0.001
>70	4.81	(3.61–6.48)	4.92	(3.50–6.90)	<0.001
CCI score (ref: 0)					
1	2.87	(1.81–4.55)	1.52	(1.24–2.12)	<0.001
≥2	2.55	(1.88–3.48)	1.85	(1.26–2.70)	<0.001
Menopausal status (ref: Postmenopausal)					
Premenopausal	1.38	(1.08–1.75)	1.00	(0.60–1.04)	0.126
HER2 (ref: Negative)					
Positive	1.51	(1.18–1.93)	0.89	(0.66–1.19)	0.508
Breast surgery (ref: Total mastectomy)					
Breast-conserving surgery	1.31	(0.86–1.68)	1.11	(0.88–1.20)	0.382
Nodal surgery (ref: SLND)					
ALND	1.28	(0.50–1.48)	1.18	(0.68–1.87)	0.492
AJCC clinical stage (ref. stage I)					
Stage II	1.81	(1.23–2.48)	1.22	(1.06–1.93)	0.003
Stage III	2.13	(1.60–2.83)	1.47	(1.13–1.85)	0.008
Hormone receptor (ref. Negative)					
Positive	0.92	(0.81–1.40)	0.90	(0.87–1.37)	0.337
Differentiation (ref: Grade I)					
Grade II	1.08	(1.02–1.36)	1.03	(1.01–1.47)	0.044
Grade III	1.12	(1.04–1.38)	1.08	(1.07–1.35)	0.013
Adjuvant chemotherapy (ref: No)					
Yes	0.73	(0.43–1.10)	0.83	(0.72–1.06)	0.361
Adjuvant radiotherapy (ref: No)					
Yes	0.77	(0.46–1.13)	0.70	(0.52–1.09)	0.304
MACE history (ref: No)					
Yes	1.16	(1.01–2.57)	1.31	(1.14–2.25)	0.005
Hyperlipidemia (ref: No)					
Yes	1.65	(1.01–2.24)	0.93	(0.61–1.51)	0.798
Hypertension (ref: No)					
Yes	1.66	(1.13–2.45)	1.13	(0.71–1.79)	0.521
Diabetes (ref: No)					
Yes	1.90	(1.35–2.66)	1.43	(0.97–2.11)	0.061
Chronic kidney disease (ref: No)					
Yes	1.28	(0.88–1.84)	1.01	(0.48–1.16)	0.174
Alcohol use (ref: No)					
Yes	1.44	(0.98–2.13)	0.98	(0.69–1.56)	0.452
Drug abuse (ref: No)					
Yes	1.39	(0.71–2.49)	0.90	(0.65–1.63)	0.833

AJCC, American Joint Committee on Cancer; CCI, Charlson comorbidity index; ALND, axillary lymph node dissection; COPD, chronic obstructive pulmonary disease; COPDAE, COPD with acute exacerbation; MACE, major adverse cardiovascular events; ref, reference group. * All covariates mentioned in Table 2 were adjusted.

## Data Availability

The data sets supporting the study conclusions are included in this manuscript.

## Abbreviations

COPD	Chronic Obstructive Pulmonary Disease
IDC	Invasive Ductal Carcinoma
COPDAE	COPD with Acute Exacerbation
aHR	Adjusted Hazard Ratio
CI	Confidence Interval
MACE	Major Adverse Cardiac Events
AJCC	American Joint Committee on Cancer
RILI	Radiation-Induced Lung Injury
RP	Radiation Pneumonitis
TCRD	Taiwan Cancer Registry Database
PSM	Propensity Score Matching
SD	Standard Deviation
AJCC	American Joint Committee on Cancer
HR	Hormone Receptor
Her-2	Human Epidermal Growth Factor Receptor-2
OS	Overall Survival
SLNB	Sentinel Lymph Node Biopsy
ALND	Axillary Lymph Node Dissection
CKD	Chronic Kidney Disease
CCI	Charlson Comorbidity Index
ICD-10-CM	International Classification of Diseases, 10th Revision, Clinical Modification
NCCN	National Comprehensive Cancer Network
GOLD	Global Initiative for Chronic Obstructive Lung Disease
